# Supplementary material for: The genome sequence of the Tenthredid wasp, Eutomostethus luteiventris (Klug, 1816) (Hymenoptera: Tenthredinidae)
Source: Wellcome Open Res. 2025 Sep 10;10:504. [Version 1] doi: 10.12688/wellcomeopenres.24855.1 (PMC12759282; doi:10.12688/wellcomeopenres.24855.1)
Supplement: Peer review history

# Reviewer report: Reviewer response for version 1

**DOI:** 10.21956/wellcomeopenres.27379.r138244

**Published:** 2026-03-02

**Authors:** Annabel Whibley

**Affiliations:** 1 The University of Auckland, Auckland, New Zealand; 2 Grapevine Improvement, Bragato Research Institute, Lincoln, New Zealand

In this data note, Halsted, Fowler and collagues report the genome assembly of the Tenthredid wasp (a type of sawfly) *Eutomostethus luteiventris. *The sample metadata protocols, sequencing and assembly pipelines and reporting template follow Darwin Tree of Life standards.  No gene annotation has been released at this time.

 Specimen identification was verified by DNA barcoding. The sample is assumed to be female, since only females have been recorded in Britain and Ireland. The reported assembly is a primary assembly only, with the genomescope2 kmer analysis showing a single peak in the profile (albeit with a slight left shoulder).  In general, the quality metrics are excellent,with > 99% of the contigs scaffolded to one of 6 pseudochromosomes. There is some ambiguity in the ordering and orientation of chromosome 5. Busco completeness is ~95%, which seems a little low but may well be typical of the taxon.  

 The recovered 30kb mitochondrial genome seems unusually large and I am a little skeptical that it is correctly assembled. *E. vegetus* has a more  typical 16.5kb mitogenome and the central portion of the contig shows (i) no homology to this sister taxon reference and (ii) strong self-self signal in a pairwise BLAST.  Some discussion of the validation of this organelle assembly would be welcome.

Are sufficient details of methods and materials provided to allow replication by others?

Yes

Is the rationale for creating the dataset(s) clearly described?

Yes

Are the datasets clearly presented in a useable and accessible format?

Yes

Are the protocols appropriate and is the work technically sound?

Yes

Reviewer Expertise:

Genomics, Bioinformatics

I confirm that I have read this submission and believe that I have an appropriate level of expertise to confirm that it is of an acceptable scientific standard.

# Reviewer report: Reviewer response for version 1

**DOI:** 10.21956/wellcomeopenres.27379.r142960

**Published:** 2026-01-21

**Authors:** Arun Arumugaperumal

**Affiliations:** 1 Department of Biotechnology, Rajalakshmi Engineering College, Thandalam, Chennai, Tamil Nadu, 602105, India

The genome sequencing project of *Eutomostethus luteiventris*, the Tenthredid wasp, has been described. This is the first report of the genome sequence of this insect. The assembly reported here is of size 272.06 Mb, spread among 6 chromosome molecules. The sex chromosomes were not identified. The mitochondrial genome has also been assembled and reported to have a size of 30.01 kb. The authors have used PacBio long-read sequencing and Hi-C sequencing data to obtain a high-quality genome assembly. The quality is evident from the high N50 values. If the annotation details were available, it could have been used to validate the quality of the assembly.

 BUSCO analysis has shown completeness of 95.9% with respect to the hymenoptera_odb10 dataset. The data note can be indexed.

Are sufficient details of methods and materials provided to allow replication by others?

Yes

Is the rationale for creating the dataset(s) clearly described?

Yes

Are the datasets clearly presented in a useable and accessible format?

Yes

Are the protocols appropriate and is the work technically sound?

Yes

Reviewer Expertise:

Bioinformatics; Genomics

I confirm that I have read this submission and believe that I have an appropriate level of expertise to confirm that it is of an acceptable scientific standard.

# Reviewer report: Reviewer response for version 1

**DOI:** 10.21956/wellcomeopenres.27379.r142964

**Published:** 2026-01-02

**Authors:** Benoit Nabholz

**Affiliations:** 1 Universite de Montpellier, Montpellier, France

The article presents a chromosome-scale assembly of *Eutomostethus luteiventris*. This represents the 24th genome published for the family Tenthredinidae.

 This work is part of the Darwin Tree of Life initiative, which produces high-quality genome assemblies, and the expected standards are met for this assembly. Interestingly, the introduction states that this species is believed to be parthenogenetic.

 Based on the specimen photograph, it appears that a female was sequenced; however, this information is not explicitly indicated in the manuscript nor in the associated databases (ENA or the DToL portal: https://portal.darwintreeoflife.org/organism/SAMEA112964468). This is consistent with the parthenogenetic nature of the species. Interestingly, GenomeScope2 and the assembly itself did not detect any heterozygosity, suggesting that parthenogenesis in this species may be associated with a loss of heterozygosity.

 Protein-coding genes have not yet been annotated, but according to the manuscript, this step will be performed in the future.

Are sufficient details of methods and materials provided to allow replication by others?

Yes

Is the rationale for creating the dataset(s) clearly described?

Yes

Are the datasets clearly presented in a useable and accessible format?

Yes

Are the protocols appropriate and is the work technically sound?

Yes

Reviewer Expertise:

Molecular evolution

I confirm that I have read this submission and believe that I have an appropriate level of expertise to confirm that it is of an acceptable scientific standard.

# Reviewer report: Reviewer response for version 1

**DOI:** 10.21956/wellcomeopenres.27379.r133005

**Published:** 2025-11-12

**Authors:** Qing-Song Zhou

**Affiliations:** 1 Chinese Academy of Sciences, Beijing, China

This paper presents a high-quality genome assembly of Eutomostethus luteiventris using PacBio HiFi and Hi-C data, which represents a significant contribution toward generating a reference genome for sawflies with high contiguity (as reflected by a high N50) and completeness (as indicated by high BUSCO scores).

 However, the authors should clarify whether the sequenced individual was female or male, as this information is essential for interpreting genome structure.

 Additionally, the reported length of the mitochondrial genome of E. luteiventris appears inconsistent with that of a congeneric species, E. vegetus (GenBank accession MT663219), which is 16,345 bp in length. The authors are encouraged to verify and, if necessary, revise the mitochondrial genome length accordingly.

 The manuscript would also benefit from additional background information on E. luteiventris, including its ecological and economic relevance, biodiversity context, and taxonomic history. Such context would help readers better appreciate the significance of this genomic resource.

 Finally, while the current assembly is already of high quality, the inclusion of Illumina short-read data for polishing and RNA-seq data for genome annotation would further enhance the accuracy and utility of this reference genome. The authors are encouraged to consider incorporating these data in future updates or supplementary analyses.

Are sufficient details of methods and materials provided to allow replication by others?

Yes

Is the rationale for creating the dataset(s) clearly described?

Yes

Are the datasets clearly presented in a useable and accessible format?

Yes

Are the protocols appropriate and is the work technically sound?

Yes

Reviewer Expertise:

Entomology, genomics, biodiversity

I confirm that I have read this submission and believe that I have an appropriate level of expertise to confirm that it is of an acceptable scientific standard.
